# Decompression Illness: Clinical Aspects of 5278 Consecutive Cases Treated in a Single Hyperbaric Unit

**DOI:** 10.1371/journal.pone.0050079

**Published:** 2012-11-21

**Authors:** Wenbing Xu, Wenwu Liu, Guoyang Huang, ZiJiao Zou, Zhiyu Cai, Weigang Xu

**Affiliations:** Department of Diving Medicine, Faculty of Naval Medicine, the Second Military Medical University, Shanghai, People’s Republic of China; St. Joseph's Hospital and Medical Center, United States of America

## Abstract

**Background:**

Decompression illness (DCI) is a major concern in pressure-related activities. Due to its specific prerequisite conditions, DCI is rare in comparison with other illnesses and most physicians are inexperienced in treatment. In a fishery area in northern China, during the past decade, tens of thousands of divers engaged in seafood harvesting and thousands suffered from DCI. We established a hyperbaric facility there and treated the majority of the cases.

**Methods and Results:**

A total of 5,278 DCI cases were admitted in our facility from February 2000 through December 2010 and treated using our recompression schedules. Cutaneous abnormalities, joint and muscular pain and neurological manifestations were three most common symptoms. The initial symptom occurred within 6 h after surfacing in 98.9% of cases, with an overall median latency of 62 min. The shorter the latent time, the more serious the symptoms would be (*P*<0.0001). Nine cases died before recompression and 5,269 were treated using four recompression schedules, with an overall effectiveness rate of 99.3%. The full recovery rate decreased with the increase of the delay from the onset of symptoms to the treatment (*P*<0.0001).

**Conclusions:**

DCI presents specific occurrence rules. Recompression should be administered as soon as possible and should never be abandoned irrespective of the delay. The recompression schedules used were effective and flexible for variety conditions of DCI.

## Introduction

The term “decompression illness” (DCI) has been used historically to refer to any medical disorder, illness, or injury arising as a result of decompression from higher to lower ambient pressure [1]. This includes decompression sickness (DCS) related to gas freed from solution in tissues during decompression and arterial gas embolism (AGE) usually caused by penetration of alveolar gas into the circulation, one of the critical complications of pulmonary barotrauma (PBT) [1]. The incidence of DCI is low in diving community and is rare in clinical practice. From 1998 to 2002, the Divers Alert Network (DAN) recorded 50,150 recreational dives, from which 28 recompressions were required, with a rate of 0.056% [2]. The mortality rate is around 10–20 diving fatalities per 100,000 DAN members [3]. Rate of occurrence of DCI in operational dives varies: typically 0.095% for commercial divers, 0.03% for US Navy divers, and 0.015% for scientific divers [4].

Herein, we report 5,278 DCI cases treated at one of our hyperbaric facilities from February 2000 through December 2010. All the patients were commercial fishery divers from an area of approximate 3,000 square kilometers in north Yellow Sea to the east of Liaotung Peninsula of China. To our knowledge, this is the DCI case series study with the largest number of cases treated in a single hyperbaric unit. The goals of this study were: (1) to observe the constitution of clinical manifestations of this case series, (2) to observe the rule of latent time from surfacing to the onset of symptoms and to analysis the correlation with severity, (3) to analysis the relationship between the delay to treatment and the outcome, and (4) to introduce our treatment table and the efficacy in treating this case series.

## Methods

### Diagnosis

Traditionally, DCI has been classified as Type 1, Type 2 DCS and AGE according to the clinical manifestations. Type 2 DCS and AGE are regarded as severe DCI and require more aggressive treatment [5]. We diagnosed DCI primarily on clinical manifestations together with the profile (the depth, bottom and decompression time) of the incident dive(s). Any symptoms arising within several hours after decompression were considered as possible DCI, especially when the safe diving limits were violated (omitted decompression, fast ascent, breath holding during ascending, etc). Some concurrent injuries during or after diving should be differentiated, such as contaminated diving gas toxicity (low quality of small compressors were frequently applied in the fishery diving), musculoskeletal strains or trauma, immersion pulmonary edema, and allergic dermatitis. The differential diagnosis can be found elsewhere [1]. The cases were classified into mild, moderate and severe categories according to the symptoms. Briefly, skin symptoms, mild to moderate musculoskeletal pain, constitutional and non-specific symptoms in the absence of obvious omitted decompression were classified as mild DCI. Severe musculoskeletal pain, mild cardiopulmonary symptoms and focal limb numbness were classified as moderate DCI. Moderate to severe cardiopulmonary and peripheral nerve symptoms, manifestations in audiovestibular and central nervous systems (CNS) were classified as severe DCI. In addition, mild or moderate symptoms in the presence of significantly missed decompression were also diagnosed as moderate or severe DCI.

### Treatment

The treatments of DCI usually include on-site management (mainly normobaric oxygen and oral fluid supplement) and recompression therapy. Due to the divers’ and the surface support personnel’s lack of basic knowledge of treatment, the on-site management was absent for all the present case series. Treatments were initiated on arrival at the hyperbaric unit.

All cases were treated with the recompression schedules developed in our department (Table 1). Mild cases were treated with Schedule 1 or 2, moderate cases with Schedule 2 or 3, and severe cases with Schedule 3 or 4. Schedule 4 is the former Soviet Union recompression treatment schedule III with minor revision [6], a schedule similar to the United States Navy (USN) Treatment Table 8 [7] and was applied in the treatment of extremely severe cases. The compression medium for all schedules was air, and oxygen was administered via face mask.

**Table 1:** Recompression schedules for the treatment of DCI.

No	Depth	Bottom time	Decompression stops (m) and the stop times (min)																					Treatment time
	(m)	(min)	54	51	48	45	42	39	36	33	30	27	24	21	18	16	14	12	10	8	6	4	2	(min)
1	30	20												3	10–20	10–20	10–20	10–20	10–20	20–30	30–60	30–90	60–90	233–413
2	40	30									2	5	5	10	20–30	20–30	20–30	20–30	20–30	30–90	30–90	30–90	60–90	323–583
3	50	30						1	4	7	7	13	13	14	20–30	20–40	20–40	40–60	40–60	60–120	90–120	90–120	90–120	590–840
4	70	30	3	3	3	3	5	5	10	15	20	25	40	60	70	110	160	180	190	210	220	240	270	1870

Note: Descent rate: 10–20 m/min; Ascent rate from bottom to first stop: 2 m/min; Travel time between stops: 1 min. Oxygen breathing begins on arrival at 18 m for 20–30 min periods separated by 5–10 min air breaks. See in Discussion for detail.

Adjuvant therapies were also adopted especially for moderate and severe cases with fluid, steroids, vitamin C, etc. Hyperbaric oxygen was recommended for all severe patients and mild to moderate patients who recovered incompletely after the first recompression, and was administered at 15 m for 80 min breathing oxygen, interrupted by 5 min air break every 20 min.

### Data Analysis

The demographic and clinical characteristics were recorded during the treatment. A retrospective analysis of all the data was performed with respect to the profile of incident dive(s), symptoms and the onset time after surfacing, delay to treatment, recompression schedules applied and the outcomes at discharging.

Approval from the Ethic Committee of the Second Military Medical University was obtained to carry out a retrospective analysis of all the data of the cases used in this study. According to the Regulation of Ethics in Clinical Study of the university, no written consent is needed in this study that referred to no personal information of patients. Verbal consent from each patient was obtained for use of their information for research. No record was made but if the patient disagreed with this, a mark of disagreement would have been written on the recording sheet of treatment of the patient and the information of this patient would have been excluded.

### Statistical Analysis

Kendall correlation analysis was used to analyze the relationship between latencies from surfacing to the onset of symptoms and severities of symptoms. The relationships between treatment outcomes and the delay to treatment or the severity were analyzed using Chi-square test. Both methods were performed using the SPSS 13 program for Windows.

## Results

### Patient Characteristics

A total of 5,587 patients were admitted and 5,278 were finally diagnosed as having DCI (Table 2). All the DCI patients were male commercial fishery divers, with the mean age 32.3±12.7 years (range: 18–55 years) and the mean years of diving 3.8±2.9 (range: one month to 15 years). 47.8% of the DCI instigating dives were performed using self-contained underwater breathing apparatus (SCUBA), and the remaining 52.2% using surface supplied diving equipment. The number of dives on the day of incident ranged from one to five with time intervals of 30–60 min. The depth was 12–30 m in 60.2% and 30–45 m in 39.3% of the dives. The bottom time was 20–60 min in 15.9% of dives, 60–120 min in 49.3% of dives, and more than 120 min in 34.8% of dives. Most of the exposures were aggressive (the depth-time was close to or exceeded limits prescribed by dive tables, but the decompression was significant shorter than acquirements) when compared with the well-known USN air diving table [7].

**Table 2:** Numbers of DCI cases with different severity.

Year	Number of cases with different severity			Total
	Mild	Moderate	Severe*	
2000	607	92	32 (4)	731
2001	521	103	39 (4)	663
2002	663	178	59 (4)	900
2003	419	121	36 (3)	576
2004	220	82	30 (3)	332
2005	418	138	21 (4)	577
2006	347	157	21 (3)	535
2007	215	91	26 (2)	332
2008	124	69	25 (2)	218
2009	158	70	17 (2)	245
2010	139	23	7 (2)	169
Total	3,831	1,124	323 (33)	5,278

* : Numbers in brackets are the fatalities which have been included in the numbers of severe cases.

### Clinical Manifestations

In the present case series, cutaneous abnormalities (rash, marbling, and swelling) were the most common symptoms and were observed in 65.1% of cases. Joint and muscular pain in the limbs was the second leading symptom and was found in 62.6% of cases. Neurological manifestations including limb numbness, muscle weakness, paresthesia, loss of consciousness, coma, hemiplegia/paraplegia, and urinary retention/incontinence were identified in 49.4% of cases. Symptoms involving cardiopulmonary system (chest pain, dyspnea, cough, hemoptysis, etc) were found in 12.7% and vestibular symptoms (auditory abnormalities, dizziness, nausea, vomiting, etc.) in 4.7% of cases. The number of patients with constitutional and nonspecific symptoms (fatigue, malaise, transient limb discomfort) in isolation was small, possibly because the divers cared little about these mild discomforts and had no idea of seeking medical services.

The numbers of mild, moderate and severe cases are listed in Table 2. Among the severe cases, 172 had spinal cord injury, of which 3 died of upper segment dysfunction; brain embolism was involved in 49 cases, of which 12 died; cardiorespiratory system was affected in 45 cases; PBT caused injury in 36 cases, of which 18 died; audiovestibular system was involved in the remaining 21 cases. Among the 221 cases of CNS DCS, spinal cord injury accounted for 77.8% (172 cases), and cerebral infarction for 22.2% (49 cases).

The latent time from surfacing to the onset of DCI symptoms is shown in Table 3. The results showed that 21.6%, 48.3%, 79.5%, 96.6%, 98.9% and 99.8% of symptoms occurred within 10 min, 30 min, 1 h, 3 h, 6 h and 24 h after surfacing, respectively. All symptoms occurred within 48 h, with a median latency of 62 min. The shorter the latency, the more severe the symptoms would be.

**Table 3:** The latent time from surfacing to the onset of symptoms and the number of cases with different severities in 5,278 DCI cases.

Latent time	Case (n)	Number of cases with different severity [n(%)]*		
		Mild	Moderate	Severe
≤10 min	1,139	545 (47.8%)	436 (38.3%)	158 (13.9%)
10–30 min	1,409	828 (58.8%)	471 (33.4%)	110 (7.8%)
30 min–1 h	1,648	1445 (87.7%)	154 (9.3%)	49 (3.0%)
1 h–3 h	905	841 (92.9%)	58 (6.4%)	6 (0.7%)
3 h–6 h	120	116 (96.7%)	4 (3.3%)	0 (0.0%)
6 h–24 h	49	48 (98.0%)	1 (2.0%)	0 (0.0%)
24 h–48 h	8	8 (100.0%)	0 (0.0%)	0 (0.0%)
Total	5,278	3,831 (72.6%)	1,124 (21.3%)	323 (6.1%)

* Kendall correlation analysis showed a weak negative correlation between the severity and the latent time (τ = −0.3604, *P*<0.0001).

### Treatment Outcomes

The fishery dives were mostly supported by small boats. Once accidents occurred, the transportation inevitably consumed at least one hour. In addition, divers had poor sense of actively seeking treatment for potential illness. Thus, although the hyperbaric facility was near in the fishing area, there was still significant delay from the onset of symptoms to the treatment, with a median delay of 9 h (range: 1–204 h) (Table 4).

**Table 4:** Delay from the onset of symptom to hyperbaric treatment and the corresponding success rate of recompression therapy.

Delay (h)	Case (n)	Complete Recovery* [n (%)]	Incomplete Recovery [n (%)]	Effectiveness [n (%)]
1–6	2,559^1^	2,401 (93.8%)	135 (5.3%)	2,536 (99.1%)^3^
6–12	1,802^2^	1,579 (87.6%)	216 (12.0%)	1,795 (99.6%)^4^
12–24	555	473 (85.2%)	80 (14.4%)	553 (99.6%)
24–36	234	189 (80.8%)	43 (18.4%)	232 (99.1%)
>36	119	90 (75.6%)	29 (24.4%)	119 (99.2%)
Total	5,269	4,732 (89.8%)	502 (9.5%)	5,234 (99.3%)

Note: Eight (note 1) and 1 (note 2) cases died before recompression were not included. Twenty-two (note 3) and 2 (note 4) death cases were in these two categories of delay, respectively. Effectiveness is the sum of the rate of complete and incomplete.

* Chi-square test showed that the complete recovery rate decreased significantly with the increase of the delay (*χ* ^2^ = 114.27, *P*<0.0001).

Table 4 also shows the relationship between the delay from the onset of symptoms to treatments and the success rate of recompression therapy at discharge. The results demonstrated that the longer the delay, the lower the rate of complete recovery (*P*<0.0001). The complete relief rate of the cases treated within 12 h following the occurrence of DCI (91.3%) was significantly higher than that of the cases treated after 24 h delay (79.0%). However, the rate of effectiveness (complete and incomplete recovery) didn’t change significantly for the cases with different delay to treatment.

As shown in Table 4 and 5, the overall effectiveness rate of recompression was 99.2% after initial recompression therapy and 99.3% at discharge. Most of the patients (5126, 97%) left the unit after the initial recompression. Sixty-two mild and 39 moderate patients who recovered incompletely after initial recompression received additional 1–5 sessions of HBO following the first treatment. All recovered completely. In 124 severe patients who improved after the first recompression, 44 received 3 to more than 100 additional sessions of HBO therapy. In which, 32 obtained complete recovery and 12 improved further. Seven out of 13 severe patients without improvement after first therapy received 3–20 sessions of HBO, 4 improved and 3 spinal cord DCS patients didn’t gain relief after 3–7 sessions of HBO and were transferred to bigger hospitals. The results were integrated in Table 5. Table 5 also shows that the more serious of symptoms, the poorer the recompression treatment outcomes would be. For the severe cases, even though the fatalities are excluded, the rate of complete recovery was only 63.8% (185/290) at discharge.

**Table 5:** Success rate of recompression therapy in 5,269 DCI cases*.

Condition	Case (n)	Complete recovery† [n (%)]		Improvement [n (%)]		Ineffectiveness△ [n (%)]		Death [n (%)]
		After initial recompression	At discharge	After initial recompression	At discharge	After initial recompression	At discharge	
Mild	3,831	3,532 (92.2%)	3,594 (93.8%)	299 (7.8%)	237 (6.2%)	0 (0.0%)	0 (0.0%)	0 (0.0%)
Moderate	1,124	914 (81.3%)	953 (84.8%)	208 (18.5%)	169 (15.0%)	2 (0.2%)	2 (0.2%)	0 (0.0%)
Severe	314	153 (48.7%)	185 (58.9%)	124 (39.5%)	96 (30.6%)	13 (4.1%)	9 (2.9%)	24 (7.6%)
Total	5,269	4,499 (87.3%)	4,732 (89.8%)	631 (12.0%)	502 (9.5%)	15 (0.3%)	11 (0.2%)	24 (0.5%)

* Nine death cases before recompression were excluded.

† The rate of complete recovery decreased significantly with the increase of severity of DCI both after initial therapy (*χ* ^2^ = 539.92, *P*<0.001) and at discharge (*χ* ^2^ = 425.48, *P*<0.001).

△ The rate of ineffectiveness increased significantly with the increase of severity both after initial therapy (*χ* ^2^ = 175.81, *P*<0.001) and at discharge (*χ* ^2^ = 114.51, *P*<0.001).

There were 11 cases with no improvement after recompression therapy, of which, nine suffered from spinal cord injury and two peripheral nerve injury, which both manifested paraesthesia in a limited skin area in calves. All cases had a marked delay before treatment.

Among the 5,269 cases receiving recompression therapy, 5 patients (0.09%) were suspected to develop CNS oxygen toxicity, with 1 presenting tonic-colonic convulsion. Four patients presented early symptoms of oxygen toxicity including paraesthesia in the lips and extremities (2 cases), facial pallor and sweating (1 case), lip twitching and dizziness (1 case). All the abnormalities occurred during or shortly after oxygen breathing at 16–12 m. At the first sign of toxicity, oxygen mask was removed and the patients breathed chamber air for 30–60 min. Oxygen breathing was resumed at the next or next two shallower stops. No compensatory lengthening of the treatment was adopted. No recurrence was observed in any of the cases. Symptoms of pulmonary oxygen toxicity, such as mild tracheal irritation similar to tracheitis of an upper respiratory tract infection, cough, chest tightness, and retrosternal pain, were not found in all the patients.

## Discussion

The 5,278 DCI cases in the present report may be by far the largest number of DCI cases treated in one hyperbaric facility during a relatively short time period. It is unlikely that a hyperbaric facility will treat so many cases in such a short period in the future. All the cases presented were finally diagnosed as DCI, and were excluded non-decompression bubble related disorders. The underlying causes of such large amount of cases include: (1) An aquaculture economic region with numerous divers who dived frequently but paid little attention to the safety regulations; (2) To increase the harvest efficiency, most of the divers ignored the principles of decompression requirements; (3) The low-quality diving equipment was improperly used and poorly maintained, resulting in a high incidence of critical failure (a broken hose, a sudden compressor failure, etc). The affected divers had to perform emergency ascents without mastering the basic breathing technique, which resulted in PBT and/or DCS; (4) Due to the characteristics of DCI that most mild to moderate symptoms will relieve or even disappear during subsequent dives, many of the diseased divers attempted to self-medicate and continue to work, which led to injury accumulation.

It is not known exactly the incidence rate of DCI for the area during the period. A survey in 2010 reported that in this area, 545 DCI cases were identified in 416,069 dives, with an incidence rate of 0.13% [8]. Of note, the fishery divers usually performed repetitive dives. A single occurrence of illness was often caused by several closely spaced dives. In addition, only the working divers were surveyed and many divers who had previously suffered from DCI had given up their diving career and were not included. These factors tend to produce an overall underestimation of DCI incidence. Furthermore, 668 divers participated in this survey [8]. If 545 cases of DCI were suffered in different divers, 81.6% of the surveyed divers had suffered DCI. Even if the cases were limited to a few divers who averaged three incidences of DCI, the proportion of divers ever suffered DCI would be as high as 27.2%.

Li et al reported 1236 DCI cases treated in their hyperbaric facility from 1995–2000 in this region [9]. The characteristics of the afflicted divers were similar with those in this report. In February 2000, our hyperbaric facility was put into service. With the advantage of the location, our facility admitted approximately 80% of the DCI cases from 2001 to 2010, with the exception of 2004 (around 50%). Therefore, it was estimated that near 7,000 DCI cases occurred in this region during the period, with an average of more than 600 cases annually. In addition, there were approximately 10–40 deaths annually during this period (anecdotal data) that died before hospitalization or at the other medical facilities in the district.

**Figure 1:**
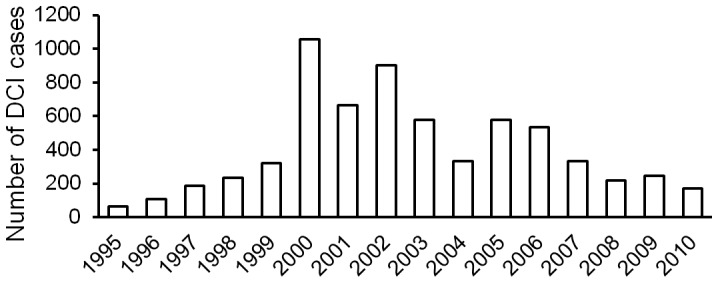
The number of DCI cases treated in two hyperbaric facilities from 1995 to 2010. The cases in 1995–1999 and a portion of the cases in 2000 were from another hyperbaric facility [9], the other portion of the cases in 2000 and the cases in 2001–2010 were treated at our facility. These two facilities received approximately 80% of DCI cases in the region during the period.

The cases reported by Li et al [9] and the cases in this report were combined in Figure 1. As shown in the figure, the number of DCI cases increased significantly after 1995 and reached a peak level during 2000–2006. The change of the policy of the local government was the primary cause of this phenomenon. The gradual easing of seafood farming policy brought a large influx of workers in this highly profitable industry with ignorance of the equally high risk. Since 2007, the local government has strengthened management on fisheries and enhanced the education on diving safety. These efforts resulted in the rapid reduction of diving injuries. It is estimated that the number of DCI cases was less than 150 in 2011 and will continue to decrease over time. The contract between our hyperbaric facility and the local hospital was terminated in January 2011, and the facility was removed.

The latency from surfacing to the occurrence of symptom mostly depends on the violation of diving rule or the severity of diving accident. Serious violations of the rule usually result in short latency [10], [11]. In the present cases, 98.9% of symptoms occurred within 6 h, with a significant shorter latency than that reported by USN (83% within 8 h) [7]. This indicated serious violations of the decompression rules by the fishery divers and thus resulted in more severe symptoms.

It is generally believed that the sooner the recompression, the better the outcome [12]. Mild initial symptoms can develop into serious manifestations, and for serious cases, delayed recompression is probably less effective [12], [13]. In this report, similar rules were clearly demonstrated. However, the effectiveness rate scarcely decreased among patients with longer delay. This suggested that hyperbaric treatment should be applied even for patients with substantially delayed presentations and improvement will most probably be obtained [14], [15].

The traditional classification of DCS (Type 1 and 2) provided a simple basis for the management of the illness among many organizations, but has practical limitations. Mild numbness in extremities would be classified as Type II and would receive intensity treatment [16]. We classify DCI into mild, moderate and severe categories depending on the severity of symptoms and decompression stress [17]. It is reasonable for different severity of DCI patients to receive corresponding intensity of recompression therapy [16]. In this study, in order to analysis the general relationship between severity of disease and other factors, CAGE due to PBT is considered one of the severe types of DCI, and differentiation from neurological DCS is usually not necessary in order not to delay the recompression [5], [18]. However, it is better to discern PBT because some special considerations such as pneumothorax should be taken during the decompression period of treatment [19]. This will be discussed in detail in separate specific study.

The outcomes of the present case series showed that adjuvant HBO following initial recompression therapy was critical for further improvement of the DCI symptoms for the patients with sequelae [20]. 98% (149/152) patients receiving additional HBO therapy recovered completely or improved significantly. Unfortunately, most of the patients chose to abort continuing treatment after the first recompression. The reasons mainly included: (1) many patients were transferred to bigger hospitals to receive comprehensive treatment, (2) many patients wouldn’t or couldn’t pay the cost of treatment, and (3) many injured divers ignored their health. If most of the patients received additional HBO therapy according to our recommendation, the success rate at discharge would have increased significantly.

The treatment schedules used in this study were initially developed in 1990’s. Schedule 1 is mainly applied for patients with mild symptoms (cutaneous manifestations, mild to moderate joint or muscle pain, constitutional and nonspecific symptoms) and delayed-treatment (>24 h) patients with mild symptoms. The total treatment time ranges from 4–7 h. If satisfactory outcome is not obtained at 30 m, treatment can progress to Schedule 2. Schedule 2 is mainly applied for patients with moderate to severe musculoskeletal pain, obvious constitutional and nonspecific symptoms, and simple “chokes”, with the total treatment time of 5.5–10 h. For pain-only patients, the effective rate is near 100%. If pain is not completely relieved when initially compressed to 40 m, further compression is usually unnecessary and adjunctive treatment such as oral administration of decongestant and anti-inflammatory drugs can be considered. Schedule 3 is mainly applied for patients with spinal cord, cerebral or vestibular DCI and those with poor response to Schedule 2. Schedule 4 is mainly used for the cases without response or with recurrence after being treated with Schedule 3. For extremely severe DCI, Schedule 4 may be directly administered. For cases of AGE secondary to PBT, Schedule 3 is preferred. However, Schedule 2 even 1 is also suitable when the manifestations of CNS are mild or the pulmonary wound is severe.

For all schedules, pure oxygen breathing begins on arrival at 18 m. The duration of oxygen breathing depends on the severity of manifestation, the response to recompression, delay to treatment, the depth-time of the incident dive(s). Some patients treated with Schedule 4 were uncooperative or unable to breathe oxygen, air breathing may be allowed throughout the recompression. Nevertheless, oxygen breathing should never be terminated prematurely and should be administered as much as possible except for toxicity considerations.

The present treatment table (including 4 schedules) is relatively concise. Each schedule is suitable for air breathing (when oxygen is not available or the equipment is not oxygen compatible), or full and partially oxygen breathing. The latter occurs when the patient is not cooperative (unable to sufficiently breathe oxygen due to the breathing resistance from facemask, weak or sleeping patients). For the patients with insufficient oxygen breathing, the procedure is to decompress them with longer stop time. Because the stop time at 18 m and shallower is not fixed, physicians need to have some experience, but the requirements are not stringent. When compared with widely used USN recompression tables, our schedules are much more concise as a whole [12].

The recovery rate of the present series (89.8%) was much higher than that reported in a review (of 1,763 cases of DCI, 80% had complete relief) [21] and in a study (of 268 patients, 86% showed complete recovery) [22]. However, immediate recompression of 166 patients achieved a complete recovery rate of 97% [21]. This further proves that early treatment favors the outcome.

The present recompression schedules are able to deal with variety conditions of DCI. However, adjuvant therapy including liquids and drugs is indispensable and critical for better outcomes, especially for severe cases [23]. For complicated cases, such as spinal cord injury and PBT, some additional rules should be followed.

One difficulty of this study was the relatively large number of patients and the limited medical staff (in most cases, only two, WBX and ZJZ) and the relative poor medical conditions. The staff members were fully engaged in the treatment of patients, near all of the patients had only physical examination and recompression treatment and other examinations such as magnetic resonance imaging, computerized tomography or neurophysiologic techniques, which could be helpful in diagnosis and evaluation of the outcomes [24], [25], were nearly absent. No autopsy was performed for all the death cases. Due to the high mobility of the divers and the lack of policy requirement, follow up after discharge was not performed. So, the long-term outcome and effectiveness is unknown. Based upon the results of physical examinations from some of the divers living in the local area, the incidence of dysbaric osteonecrosis (DON), one of the main sequelae of DCI, is increasing with an estimate of higher than 20%. Epidemiological studies are warranted for more detailed information in the future.
